# Transmission distortion and genetic incompatibilities between alleles in a multigenerational mouse advanced intercross line

**DOI:** 10.1093/genetics/iyab192

**Published:** 2021-11-15

**Authors:** Danny Arends, Stefan Kärst, Sebastian Heise, Paula Korkuc, Deike Hesse, Gudrun A Brockmann

**Affiliations:** Breeding Biology and Molecular Genetics, Albrecht Daniel Thaer Institute for Agricultural and Horticultural Sciences, Humboldt University Berlin, Berlin D-10115, Germany; Breeding Biology and Molecular Genetics, Albrecht Daniel Thaer Institute for Agricultural and Horticultural Sciences, Humboldt University Berlin, Berlin D-10115, Germany; Breeding Biology and Molecular Genetics, Albrecht Daniel Thaer Institute for Agricultural and Horticultural Sciences, Humboldt University Berlin, Berlin D-10115, Germany; Breeding Biology and Molecular Genetics, Albrecht Daniel Thaer Institute for Agricultural and Horticultural Sciences, Humboldt University Berlin, Berlin D-10115, Germany; Breeding Biology and Molecular Genetics, Albrecht Daniel Thaer Institute for Agricultural and Horticultural Sciences, Humboldt University Berlin, Berlin D-10115, Germany; Breeding Biology and Molecular Genetics, Albrecht Daniel Thaer Institute for Agricultural and Horticultural Sciences, Humboldt University Berlin, Berlin D-10115, Germany

**Keywords:** intergenerational effects, non-Mendelian inheritance, genetic incompatibilities, interactions, allele transmission bias

## Abstract

While direct additive and dominance effects on complex traits have been mapped repeatedly, additional genetic factors contributing to the heterogeneity of complex traits have been scarcely investigated. To assess genetic background effects, we investigated transmission ratio distortions (TRDs) of alleles from parent to offspring using an advanced intercross line (AIL) of an initial cross between the mouse inbred strains C57BL/6NCrl (B6N) and BFMI860-12 [Berlin Fat Mouse Inbred (BFMI)]. A total of 341 males of generation 28 and their respective 61 parents and 66 grandparents were genotyped using Mega Mouse Universal Genotyping Arrays. TRDs were investigated using allele transmission asymmetry tests, and pathway overrepresentation analysis was performed. Sequencing data were used to test for overrepresentation of nonsynonymous SNPs (nsSNPs) in TRD regions. Genetic incompatibilities were tested using the Bateson–Dobzhansky–Muller two-locus model. A total of 62 TRD regions were detected, many in close proximity to the telocentric centromere. TRD regions contained 44.5% more nsSNPs than randomly selected regions (182 *vs* 125.9 ± 17.0, *P* < 1 × 10^−4^). Testing for genetic incompatibilities between TRD regions identified 29 genome-wide significant incompatibilities between TRD regions [*P*_(BF)_ < 0.05]. Pathway overrepresentation analysis of genes in TRD regions showed that DNA methylation, epigenetic regulation of RNA, and meiotic/meiosis regulation pathways were affected independent of the parental origin of the TRD. Paternal BFMI TRD regions showed overrepresentation in the small interfering RNA biogenesis and in the metabolism of lipids and lipoproteins. Maternal B6N TRD regions harbored genes involved in meiotic recombination, cell death, and apoptosis pathways. The analysis of genes in TRD regions suggests the potential distortion of protein–protein interactions influencing obesity and diabetic retinopathy as a result of disadvantageous combinations of allelic variants in *Aass*, *Pgx6*, and *Nme8*. Using an AIL significantly improves the resolution at which we can investigate TRD. Our analysis implicates distortion of protein–protein interactions as well as meiotic drive as the underlying mechanisms leading to the observed TRD in our AIL. Furthermore, genes with large amounts of nsSNPs located in TRD regions are more likely to be involved in pathways that are related to the phenotypic differences between the parental strains. Genes in these TRD regions provide new targets for investigating genetic adaptation, protein–protein interactions, and determinants of complex traits such as obesity.

## Introduction

Over the last two decades genome wide association studies (GWAS) have identified a virtual avalanche of genetic variants associated with complex phenotypes and diseases (Buniello *et al.* 2019). However, although numerous, these identified genetic variations only partially explain the observed heritability in complex phenotypes either individually or combined (Zuk *et al.* 2012). This so-called “missing heritability” problem has been observed for many complex phenotypes and diseases, such as obesity which has long been known as a major risk factor for many diseases in the later course of life (Kopelman 2007; Tremmel *et al.* 2017). For obesity, as well as other complex phenotypes, a great amount of effort was spent finding genetic determinants (Willer *et al.* 2009; Speliotes *et al.* 2010). As a result, a large number of genetic variants contributing to obesity have been identified (www.genome.gov/gwastudies). However, most loci found by GWAS had small effects (Willer *et al.* 2009; Shungin *et al.* 2015). For example, the 97 significant loci identified for body mass index (BMI), accounted for only 2.7% of the corresponding variation (Locke *et al.* 2015).

One of several reasons discussed for the “missing heritability” problem in GWAS is the genetic heterogeneity of loci contributing to complex traits among the individuals in populations (Heid *et al.* 2010; Shungin *et al.* 2015). In addition to direct genetic effects, substantial phenotypic variation among individuals can be caused by preferential allele combinations or by allele incompatibilities in the genome of individuals. Allele incompatibilities are combinations of two (or more) alleles which when inherited together cause a disadvantage for the individual. Inheriting the disadvantageous allele combination leads to a survival disadvantage for this individual (*e.g.*, less vigor, less successful reproduction). Allelic interactions leading to such disadvantages can be detected as transmission ratio distortion (TRD) from parent to offspring (also called allele transmission bias). Although TRD has been widely reported in a wide range of species (Lyon 2003; Huang *et al.* 2013b; Li *et al.* 2019), its functional impact on complex phenotypes has rarely been studied.

To investigate genetic background effects such as TRD, we examined three generations (26, 27, and 28) of an advanced intercross line (AIL) between two inbred mouse lines, the Berlin Fat Mouse Inbred (BFMI) line and the C57BL/6NCrl (B6N) line. In AIL populations from two inbred founders, only two parental alleles can segregate at each locus, making the population heterogeneous but less complex than human populations. Therefore, an AIL population is well suited to study deviations from Mendelian inheritance.

The BFMI is an inbred line generated from an outbred population descending from several different founder mice bought at pet shops across Berlin. The BFMI line had been selected for high fatness for more than 100 generations before it was inbred (Wagener *et al.* 2006). Unfortunately, the original founders of the BFMI do not exist anymore. However, whole-genome DNA sequencing data showed that the BFMI genome is a mixture of *Mus musculus musculus* and *Mus musculus domesticus*.

Recently, a major recessive mutation responsible for the juvenile obesity phenotype (*jObes1*) in BFMI mice was fine-mapped to a 370 kb region on chromosome 3 (Arends *et al.* 2016). Complementation tests suggested *Bbs7* as the most likely causal gene in this region. The *jObes1* locus accounted for around 40% of the body weight variance (Neuschl *et al.* 2010; Arends *et al.* 2016), while environmental effects accounted for 34%. Hence, around 26% of the variance in body weight is still unexplained.

During the process of long-term selection for a phenotype (*e.g.*, high fatness), enrichment or even fixation of alleles that positively contributed to the selection response have been observed (Hirsch *et al.* 2014). Complimentary, the frequency of conflicting alleles impairing fitness, survival of gametes or embryos would be expected to be reduced or lost in the process of selection. In particular in complex traits, such as obesity, where many genes with diverse allelic variants contribute to the phenotype, the compatibility of interacting alleles is expected to be a driving force for the selection response. Therefore, long-term selection can be considered as coevolution of alleles during the process of adaptation to selection pressure, environment, and genetic background.

The same principles of shaping the genomic composition occurred during the inbreeding history of every inbred mouse line, including BFMI and B6N, which were used in our experiment. Experimental inbreeding usually starts with several full-sib families (Flurkey *et al.* 2009). During the process of repeated mating of full-sibs, when the genome gets more and more reduced to one haplotype, some inbred families go extinct because of direct lethal recessive allele effects, lethal combinations of alleles across the genome, or the inbred family collapses because of low vigor or insufficient reproduction (Whitlock 2000; Zajitschek *et al.* 2009; Fitzpatrick and Evans 2009) eventually as a result of genomic incompatibility. However, low level incompatibilities, which do not directly cause lethality or affect fertility might be retained, invisible, inside an inbred line. During the inbreeding process haplotypes get reduced, and incompatibilities might survive since there is no choice of alternative allele anymore. In the end, a kind of optimized genome remains alive as established inbred strain. 

## Materials and methods

### Mouse population

A total of 348 male mice of an AIL in generation 28 as well as their 62 parents and 66 grandparents from generations 27 and 26 were genotyped. The AIL population originates from the mapping population of a cross between a male mouse of the obese line BFMI860-12 (BFMI) and a female of the lean line C57BL/6NCrl (B6N), that had been initially used to map the juvenile obesity locus *jObes1* (Neuschl *et al.* 2010). Beginning in generation F_1_, individuals were randomly mated to mice from the same generation using the program RandoMate (Schmitt *et al.* 2009).

### Husbandry conditions

All experimental treatments of animals were approved by the German Animal Welfare Authorities (approval no. G0016/11). All mice were maintained under conventional conditions and a 12:12 h light:dark cycle (lights on at 6:00am) at a temperature of 22 ± 2°C. Animals had *ad libitum* access to food and water. To perform fine mapping of the obesity quantitative trait locus (QTL; Arends *et al.* 2016), generation 28 was fed with a rodent high-fat diet containing 19.5 MJ/kg of metabolizable energy, 45% from fat, 24% from protein, and 31% from carbohydrates (E15103-34, ssniff EF R/M; Ssniff Spezialdiäten GmbH, Soest, Germany). All other generations used in this study were fed a standard breeding diet (V1534-000, ssniff EF R/M; Ssniff Spezialdiäten GmbH, Soest, Germany).

### Genotypes

Genotypes were generated at GeneSeek (Lincoln, NE, USA) using the Mega Mouse Universal Genotyping Array. These arrays are SNP genotyping arrays based on the Illumina Infinium platform designed by investigators at the University of North Carolina at Chapel Hill, manufactured by Illumina (San Diego, CA, USA), and distributed by Neogen Inc (Lansing, MI, USA) (Morgan and Welsh 2015). This array contains probes targeting 77,800 known SNPs. SNP probes were remapped to the reference genome (GRCm38.p6) using BLASTN with default settings (Camacho *et al.* 2009). To increase the certainty of genotype calls, genotypes with a GenCall score greater than 0.7 were considered confidently called, although the manufacturer’s recommendation is a GenCall score > 0.15. SNPs that mapped to multiple positions in the genome, noninformative SNPs, and SNPs with genotype call rates below 90% were removed from further analysis. In total, 14,415 highly confident SNPs passed all quality checks and were informative between BFMI and B6N. Marker density, as well as minor allele frequencies within and outside of TRD regions were visualized and can be found in Supplementary Table S5.

Furthermore, checking parent–child relations in our trio data identified three individuals in generation 28 where one of the parents was wrongly assigned, these three individuals were removed from further analysis. Similarly, one individual in generation 27 was found to have a wrong parent assignment, leading to the removal of this individual and its four offspring in generation 28. Phasing of the heterozygous genotypes of the AIL animals of generation 28 toward the parental population (generation 27), and of generation 27 to generation 26 was done using Beagle v4.1 (Browning and Browning 2007) with standard settings. Raw and phased genotypes of all individuals that passed quality control [*N*_(28)_ = 341, *N*_(27)_ = 61, and *N*_(26)_ = 66], the genetic map, and pedigree data are available in Supplementary Table S2.

### Allele transmission from heterozygous parents

Deviations from expected Mendelian inheritance ratios are named TRD. Such deviations have been commonly observed in experimental crosses as well as in natural populations. We used an extension of the transmission asymmetry test and parental asymmetry test to detect parent-of-origin dependent effects on the frequency of the transmission of a specific SNP allele from parent to offspring using trios in our AIL design (Weinberg *et al.* 1998). For example: To determine if one of the alternative paternal alleles (*e.g.*, A *vs* B allele) at a SNP locus is inherited more often than expected by Mendel (50%), pups were analyzed in generation 28 of fathers (generation 27) that were heterozygous for this SNP. We only tested markers at which at least 10 heterozygous fathers (or mothers) were available. We counted the number of offspring where a specific paternal allele was transmitted. When both parents were heterozygous, the allele transmitted cannot be determined and this transmission was not counted in the test statistic. Furthermore, markers were tested for Hardy–Weinberg equilibrium (HWE) using the code developed by Wigginton *et al.* (2005). Markers not in HWE were excluded, since the *χ*^2^ test for TRD is only valid when a marker is in HWE. A *χ*^2^ test was used to test if this distribution of paternally inherited alleles significantly deviated from the expected Mendelian inheritance ratios (**Pat**), and similarly for maternally inherited alleles (**Mat**).

***Pat***: Analysis of allele TRD from heterozygous fathers to offspring

*χ* ^2^_Pat_ = (*P*_AB_ – *P*_BA_)^2^/(*P*_AB_ + *P*_BA_)

***Mat***: Analysis of allele TRD from heterozygous mothers to offspring

*χ* ^2^_Mat_ = (*M*_AB_ – *M*_BA_)^2^/(*M*_AB_ + *M*_BA_)

*χ* ^2^ scores were transformed into *P*-values using the appropriate conversions and then transformed into LOD scores using –log_10_(*P*-value). A total of 5% and 1% significance thresholds were determined by Bonferroni correction (5% ≥ 6.75, 1% ≥ 7.45). Significant regions were defined as the region from the first to the last flanking marker above the 1% significance threshold (LOD scores ≥ 7.45).

### Genetic variants in TRD regions

Parental genomes (BFMI860-12 and B6N) were paired-end sequenced using the “Illumina HiSeq” platform (Illumina Inc., San Diego, CA, USA). Obtained DNA reads were trimmed using trimmomatic (Bolger *et al.* 2014) after which trimmed reads were aligned to the mouse genome (mm10, GRCm38.p6) using the Burrows–Wheeler Aligner software (Li and Durbin 2009). The subsequent SAM files were converted to BAM files, sorted, and indexed using Samtools (Li *et al.* 2009; Morgan *et al.* 2017). (Optical) Duplicate reads were removed using Picard tools v2.19.0 (McKenna *et al*. 2010), after which indel realignment and base recalibration was done using the GATK v4.1.0.0 (McKenna *et al.* 2010), according to GATK best practices (McKenna *et al*. 2010). Sequence variants were called using BCFtools (Morgan *et al.* 2017) Variants passing quality control were further annotated using the Ensembl Variant Effect Predictor (McLaren *et al.* 2016). DNA sequencing data allowed to identify nonsynonymous SNPs (nsSNPs) in genes located in TRD regions between the founding strains.

A permutation strategy was used to detect over- and/or underrepresentation of nsSNPs in the regions showing TRD. We performed 50,000 permutations, each time drawing 1424 protein-coding genes at random, not allowing duplicate genes or selection of predicted genes (GM/RIKEN). For every permutation, the number of nsSNPs and the number of genes with nsSNPs was recorded. After 50,000 permutations, a distribution of the total number of nsSNPs (and genes) in the random data was obtained, which was compared with the observed data.

### Pathway overrepresentation analyses

We extracted all protein-coding genes inside the significant regions using biomaRt (Kasprzyk 2011) for each of the different types of allele TRD: Preferred paternal transmission of the BFMI allele (Pat_BFMI), preferred paternal transmission of the B6N allele (Pat_B6N), preferred maternal transmission of the BFMI allele (Mat_BFMI), and preferred maternal transmission of the B6N allele (Mat_B6N). To identify potential functional clustering of genes within one of these groups, pathway overrepresentation analyses was performed using innateDB (Breuer *et al.* 2013) with KEGG (Ogata *et al.* 1999) and Reactome (Joshi-Tope *et al.* 2005) as the pathway providers. Overrepresentation was tested using a hypergeometric test. *P*-values reported for pathway overrepresentation were Benjamini–Hochberg corrected [*P*_(BH)_; Benjamini and Hochberg 1995]; *P*_(BH__)_ < 0.05 were considered significant. Pathway analysis was additionally performed with genes showing nsSNPs using the same grouping as before, with “_SNP” added to the group identifier (Pat_BFMI_SNP, Mat_BFMI_SNP, Pat_B6N_SNP, and Mat_B6N_SNP).

### Genetic incompatibilities

Testing for pairwise genetic incompatibilities in an exhaustive manner is not advisable because of the large number of statistical tests required for 20k SNP markers leading to a severe multiple testing correction. Our hypothesis is that genetic incompatibilities cause allele TRDs. For testing incompatibilities, 3 × 3 contingency tables were created using the top SNP marker in the TRD region 1 (M1) *vs* the top SNP marker in TRD region 2 (M1), and the number of co-occurrences between different alleles was counted. If no top marker was present in a region *e.g.*, Pat_R5, due to all markers showing a similar distortion, the proximal flanking marker was used as top marker. Our method for scoring genetic incompatibility is very similar to the methods used by Ackermann and Beyer (2012) and Corbett-Detig *et al.* (2013). A 3 × 3 table of expected co-occurrences based on the observed allele frequencies at markers M1 and M2 was generated assuming independent segregation of each marker. Resulting *χ*^2^ scores were transformed into *P*-values, which are then transformed to LOD scores as described before. For each pair of markers that showed a genome-wide significant interaction [*P*_(BF)_ < 0.05], founder alleles of the group which shows the most reduction (in percentage) between observed and expected co-occurrences was used for the visualization seen in Figure 2.

Pairwise interactions tests were only performed between detected TRD regions, while correction for multiple testing was done using genome-wide thresholds. This first involved estimating the number of effective tests by using the simpleM method (Gao 2011). The simpleM method was designed to estimate the number of independent tests in a GWAS by considering linkage between markers. The simpleM procedure estimated 1008 independent test (at a fixLength of 1200) which is much lower than the number of genetic markers. This reduction in total tests can be explained by strong linkage between markers in our AIL population. LOD thresholds were adjusted for multiple testing using Bonferroni correction [*P*_(BF)_] and the number of independent tests estimated with the simpleM method (*n* = 1008). Since we tested pairwise but without repeating the test for a pair we have already tested, the number of tests had to be multiplied with itself and reduced by half leading to LOD scores calculated as: −log10[threshold/(1008 * 1008 * 0.5)]. Dependent on the significance threshold, this leads to the following genome-wide adjusted LOD thresholds: significant if LOD > 7.0 [*P*_(BF)_ < 0.05] and highly significant if LOD > 7.7 [*P*_(BF)_ < 0.01].

We then continued our investigation of known protein–protein interactions between genes with nsSNPs within these regions of incompatibility using the Search Tool for the Retrieval of Interacting Genes/Proteins (STRING) database version 11 (Szklarczyk *et al.* 2019). In total, 9,602,772 known physical protein–protein interactions for *M.* *musculus* (SpeciesID 10090) are listed in this database (10090.protein.physical.links.v11.0). We first only considered the protein–protein interactions between the 128 genes with one or more nsSNP(s). Afterwards, we overlaid the gene location data with the TRD regions for which we found genome-wide significant evidence of genetic incompatibilities. This was done to see if identified genetic compatibility could be explained by known physical protein–protein interactions in which both participating genes show one or more nsSNP(s).

## Results

### Allele transmission ratios from heterozygous parents to offspring

The probability for the transmission of parental alleles to their offspring can be calculated according to Mendelian laws. Deviations from those expected inheritance patterns might have genetic reasons that we intend to identify. To test for TRDs, we used all 341 males of generation 28 of the AIL and tested how their parents (generation 27) transmitted their alleles to this generation. TRD was detected for 62 genomic regions at a genome-wide Bonferroni corrected significance level of 0.01. These regions can be grouped by the preferentially transmitted allele based on the parental origin (paternal/maternal) and the founder strain origin (B6N/BFMI). Significant paternal allele TRD was detected for 1068 out of 18,114 tested SNPs. Paternally affected TRD of SNPs clustered into 31 chromosomal regions, due to linkage between neighboring SNPs. For maternal TRD, 1138 SNPs located in 31 regions were found (Supplementary Table S1). Overlaying the paternal and maternal TRD regions showed that 14 regions showed both paternal and maternal TRD. In overlapping TRD regions always the same founder allele of either mouse strain B6N or BFMI was preferentially transmitted.

**Figure 1:**
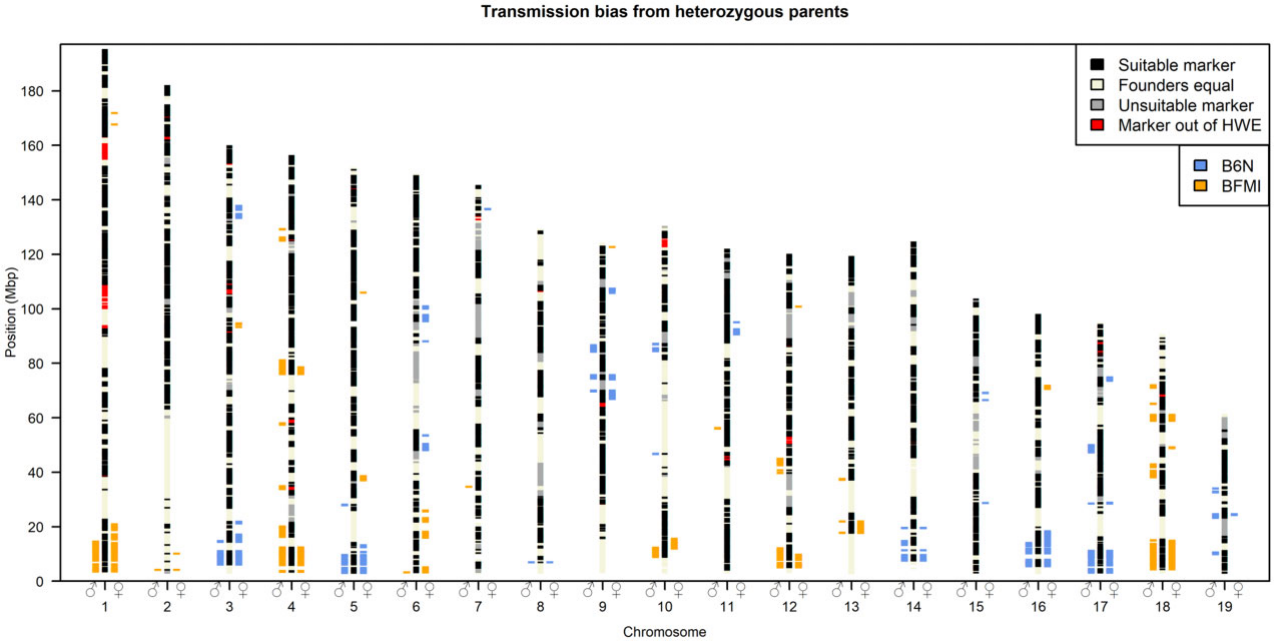
Genomic regions showing allele TRD toward generation 28. Bars left of the chromosomes mark the SNPs which show paternal TRD (♂); bars on the right side show maternal TRD (♀), using a genome-wide significance level of *P* < 0.01. Colors show the origin of the allele preferentially transmitted, blue: B6N allele, orange: BFMI allele. Chromosomal black areas (suitable markers) contain markers which passed quality control steps, segregate between the founder lines (BFMI and B6N), and have at least 10 heterozygous parents in generation 27 required to perform a valid *X*^2^ test. Chromosomal beige areas (Founders equal) are markers at which the BFMI and B6N have the same allele, these markers do not segregate in the AIL population, and cannot be tested for TRD. Chromosomal gray areas (unsuitable markers) have not been tested due to lack of heterozygous parents in generation 27 at these markers. Chromosomal red areas are not in HWE in generation 28, since HWE is an assumption underlying a valid TRD test, these areas were excluded from TRD analysis.

TRD was detected consistently across large genomic regions, in which a high number of markers showed the same transmission bias for one of the two founder alleles B6N or BFMI. The 19 regions showing TRD supported by at least 50 markers are shown in Table 1, all detected TRD regions and their observed transmission distortions are summarized in Supplementary Table S1, and visualized in Figure 1. Genotypes, genetic map, and pedigree of the AIL individuals can be found in Supplementary Table S2.

**Table 1:** TRD from paternal and maternal side, supported by at least 50 SNPs (*P* < 0.01) per identified region

Region ID	Chr	Proximal	Top	Distal	Region size	nSNPs	Preferred allele	Transmissions region average (SD)		Top marker	Transmission top marker		BFMI allele distortion (%)
								BFMI	B6N		BFMI	B6N	
Pat_R1	1	3,668,628	11,643,615	14,698,538	11,029,910	68	BFMI	146.1 (16.3)	41.1 (6.6)	UNC109624	152	30	67.0
Pat_R3	4	3,569,913	6,093,982	12,555,306	8,985,393	83	BFMI	168.0 (5.0)	55.3 (3.9)	UNC6664886	169	48	55.8
Pat_R7	4	76,193,199	78,074,351	81,245,565	5,052,366	58	BFMI	79.6 (9.0)	15.8 (3.0)	UNC7556251	84	15	69.7
Pat_R17	12	5,253,913	8,416,509	12,177,986	6,924,073	92	BFMI	163.3 (28)	53.5 (9.6)	UNC20594325	207	47	63.0
Pat_R21	16	5,617,528	14,151,479	14,151,479	8,533,951	93	B6N	31.5 (7.2)	147.9 (15.1)	UNC26373573	35	177	−67.0
Pat_R22	17	3,264,958	5,991,544	11,318,508	8,053,550	77	B6N	34.0 (16.2)	99.4 (27.1)	UNC170286629	16	139	−79.4
Pat_R23	17	47,490,686	48,644,966	50,134,302	2,643,616	50	B6N	41.7 (30.8)	117.1 (10.1)	UNC27963988	19	110	−70.5
Pat_R24	18	4,516,519	12,242,865	13,902,153	9,385,634	71	BFMI	158.7 (2.9)	61.4 (3.3)	UNC28742422	155	56	46.9
Pat_R25	18	38,281,545	38,975,190	42,987,483	4,705,938	54	BFMI	168.7 (4.1)	54.2 (9.3)	UNC29080241	168	46	57.0
Mat_R1	1	3,668,628	3,668,628	21,099,704	17,431,076	94	BFMI	136.2 (17.8)	56.9 (10.3)	UNC010515443	178	69	44.1
Mat_R2	3	6,274,425	11,119,314	17,230,305	10,955,880	53	B6N	24.9 (13.5)	129.2 (5.8)	backupUNC030002827	13	126	−81.3
Mat_R5	3	133,540,430	136,529,917	137,921,098	4,380,668	52	B6N	35.4 (4.1)	105.3 (12.8)	UNC6326383	33	113	−54.8
Mat_R6	4	3,569,913	3,918,966	12,555,306	8,985,393	83	BFMI	144.5 (5.5)	50.1 (6.4)	UNC6640040	135	38	56.1
Mat_R12	6	48,276,599	48,276,599	53,750,577	5,473,978	53	B6N	24.8 (5.0)	80.5 (15.4)	UNC11030573	27	96	−56.1
Mat_R15	9	67,022,716	74,576,994	75,773,843	8,751,127	54	B6N	37.3 (12.9)	110.8 (16.1)	UNC090145124	23	99	−62.3
Mat_R16	9	106,017,492	106,605,721	107,516,430	1,498,938	58	B6N	29.3 (4.5)	87.1 (2.4)	UNC17077906	27	87	−52.6
Mat_R23	16	5,617,528	5,617,528	18,450,764	12,833,236	163	B6N	23.5 (8.7)	108.4 (11.2)	UNC160000883	10	130	−85.7
Mat_R25	17	3,264,958	5,991,544	11,318,508	8,053,550	77	B6N	20.0 (4.4)	106.8 (22.4)	UNC170286629	20	164	−78.3
Mat_R28	18	4,516,519	8,317,246	15,000,978	10,484,459	81	BFMI	170.6 (19.5)	52.0 (2.8)	UNC28690832	50	197	−59.5

Region ID, the identifier of the region Pat_R# stands for paternal TRD and Mat_R# stands for maternal TRD within the region; Chr, Chromosome on which the distortion was detected; Proximal, Top, and Distal = start, top, and end positions of the region on the chromosome (based on the GRCm38.p6/mm10 genome); nSNPs, number of SNP markers on the array that support the TRD region; preferred allele = the allele preferentially transmitted; average transmission counts for founder alleles across all markers in the region are listed with their standard deviation in brackets. Top marker as well as transmission at the top marker and BFMI allele distortion (%) is shown in the last four columns. See Supplementary Table S1 for an overview of all 31 paternal and 31 maternal regions. The observed very small standard deviations in almost all regions indicate that the distortion observed is consistent across the regions identified.

As an example, Pat_R3 is a region on chromosome 4 from 3.5 to 12.5 Mb, which showed paternal TRD of the BFMI allele supported by 83 markers. In the AIL population, we observed at each marker around 217 paternal allele transmissions from generation 27 to 28, meaning we expect 108.5 transmissions of the BFMI, as well as 108.5 transmissions of the B6N allele. However, in this region markers on average showed 168 (±5.0) transmissions of the BFMI allele from heterozygous fathers to their offspring, while the B6N allele was only transmitted 55.3 (±3.9) times. At the top marker in this region on chromosome 4 (UNC6664886), we observed 169 BFMI *vs* 48 B6N transmissions. This means that transmission of the BFMI allele was observed 55.8% more often than expected by Mendelian inheritance, the likelihood of this happening was estimated by *χ*^2^-test to be lower than 1 × 10^−14^.

When performing the same tests for the allele transmission from AIL generation 26 to 27 [transmission from grandparents (*n* = 66) to parents (*n* = 61)], where sample sizes were much smaller, we relaxed our threshold for significance to *P* < 0.05. In generation 26 to 27, 0 and 38 SNPs still showed significant paternal or maternal TRD, respectively. The overlap between SNPs detected in generations 26 to 27 *vs* 27 to 28 was 100%, meaning that all TRD seen from generation 26 to 27 was also found (much more significant) from generations 27 to 28.

In our population, we observed that the TRD is a local event affecting many SNPs in a well-defined chromosomal region due to linkage between neighboring SNPs. Since, many recombinations have accumulated over 28 generations of mating, the length of the TRD affected regions is between 41.1 kb and 17.4 Mb (Supplementary Table S1). In these regions, SNPs that showed TRD are tightly linked (Figure 1), which can be seen by TRD SNPs clustering together into regions. Very small standard deviations of averaged TRD transmissions (Table 1) were observed for all regions, which indicates that distortion observed was consistent across the TRD region. This was further supported by the observation that all SNPs in a certain region always showed the same direction of transmission toward one of the alleles from the original founder strains of the AIL population. For example, Mat_R1 showed the BFMI allele was preferentially transmitted in the maternally distorted region on chromosome 1 between 3.7 and 21.1 Mb. For the 94 markers in this region, we observed 136.2 ± 17.8 transmissions of the BFMI allele *vs* 56.9 ± 10.3 transmissions of the B6N allele from mother generation 27 to offspring (generation 28).

Interestingly, regions showing significant TRD on 10 out of 19 autosomes (autosomes 1, 3, 4, 5, 10, 12, 14, 16, 17, 18) are located close to the telocentric centromere (Figure 1). These 10 telocentric centromere regions showed both paternal and maternal TRD with a consistent preference of the founder allele. While these telocentric centromere regions showed TRD for both paternal and maternal alleles, we observed that nontelocentric centromere regions (*e.g.*, Mat_R12, Table 1) tend to show TRD only when inherited from either the paternal or the maternal side.

### Genetic variants in TRD regions

To identify candidate genes for each region, and to investigate possible causes for the observed TRD, protein-coding genes located in TRD regions were examined. Sequence variants were detected by comparing the BFMI sequence to the B6N reference genome (ENSEMBL, GRCm38.p6) (Supplementary Table S3). In the 62 identified TRD regions, 1167 unique protein coding genes were located. In detail, these were 292 genes in Pat_BFMI regions, 362 in Mat_BFMI, 335 in Pat_B6N, and 567 in Mat_B6N. Among those, 389 genes were overlapping between paternal and maternal TRD regions.

In the 1167 unique protein coding genes located in 62 TRD regions, 182 nsSNPs were found located in 128 (10.9%) genes. Permutation analysis showed that the density of nsSNPs in the TRD regions was 1.445 times higher than expected from random distribution. Results from 50,000 permutations showed an average of 125.9 ± 17.0 (SD) nsSNPs per 1167 randomly selected genes with a maximum value of 180 SNPs observed during permutation. These results provide evidence that nsSNPs are significantly overrepresented in TRD regions (*P* < 1 × 10^−4^).

### Pathway overrepresentation analysis

Pathways analysis was performed twice, once we investigated pathway overrepresentation by including all protein-coding genes in the specified TRD regions, followed by only investigating genes that carry nsSNPs.

Analysis of all genes in TRD regions with higher transmission of the paternal BFMI allele (Pat_BFMI) showed slight but significant pathway overrepresentation of the three pathways “Post-transcriptional silencing by small RNAs” [*P*_(BH)_ = 0.008], “Small interfering RNA (siRNA) biogenesis” [*P*_(BH)_ = 0.011], and “MicroRNA (miRNA) biogenesis” [*P*_(BH)_ = 0.016] (Supplementary Table S4—Pat_BFMI for the full list).

All genes located in maternal inherited (Mat_BFMI) showed highly significant overrepresentation for pathways such as “DNA methylation” [*P*_(BH)_ < 5.7 × 10^−16^], “Meiotic Recombination” [*P*_(BH)_ < 6.61 × 10^−15^], “Packaging Of Telomere Ends” [*P*_(BH)_ < 1.70 × 10^−13^], “Chromatin organization” [*P*_(BH)_ < 2.66 × 10^−10^] and “Deposition of new CENPA-containing nucleosomes at the centromere” [*P*_(BH)_ < 3.67 × 10^−10^; Supplementary Table S4—Mat_BFMI for the full list]. All these pathways are involved in chromosome stability/maintenance as well as centromere and nucleosome organization. We also found strong overrepresentation of the “Signaling by Wnt” [*P*_(BH)_ = 1.09 × 10^−5^] pathway, as well a weak overrepresentation of the “Retinol metabolism” [*P*_(BH)_ = 0.010] pathway. These are two interesting pathways in the context of BFMI mice, which will be elaborated in more detail in the discussion section.

No strong overrepresentation or overlap was found for all genes located in paternal/maternal B6N TRD regions (Supplementary Table S4—Pat_B6N and Mat_B6N). Only three pathways were found weakly overrepresented when using all genes from maternal B6N (Mat_B6N) TRD regions: “Nitrogen metabolism” [*P*_(BH)_ < 0.019], “Reversible hydration of carbon dioxide” [*P*_(BH)_  < 0.033], and “Osteoclast differentiation” [*P*_(BH)_ < 0.042].

If we focused on genes with nsSNPs in TRD regions, where the BFMI allele is preferentially passed by the father (Supplementary Table S4—Pat_BFMI_SNP), no strong pathway overrepresentation was observed. Only two pathways reach significance after Benjamini–Hochberg correction: “S Phase” [*P*_(BH)_ = 0.015] and “Extracellular matrix organization” [*P*_(BH)_ = 0.025]. However, the number of genes found in TRD regions (two for both) *vs* the total number of genes annotated to these pathways make this overrepresentation very weak (117 and 216, respectively). If we examined the genes with nsSNPs from TRD regions of paternal B6N allele transmission (Supplementary Table S4—Pat_B6N_SNP), “Interferon Signaling” [*P*_(BH)_ = 0.016], “Cell cycle” [*P*_(BH)_ = 0.023], and “Metabolism of lipids and lipoproteins” [*P*_(BH)_ = 0.023] were weakly significantly overrepresented. Again, the numbers of genes in TRD regions is small compared to the total number of genes annotated to these pathways.

Genes with nsSNPs from TRD regions of the maternal BFMI allele (Supplementary Table S4—MAT_BFMI_SNP) showed only one very weak significant pathway overrepresentation: “Extracellular matrix organization” [*P*_(BH)_ = 0.046], which was also found for genes with nsSNPs in TRD regions where the BFMI allele was preferentially passed by the father (Pat_BFMI_SNP). Genes with nsSNPs in TRD regions of the maternal B6N allele (Supplementary Table S4—Mat_B6N_SNP) also showed overrepresentation of multiple pathways overlapping with pathways found for genes with nsSNPs in paternal B6N regions (“Cell cycle,” “Interferon Signaling,” “Metabolism,” and “Metabolism of lipids and lipoproteins”). Furthermore, maternal genes with nsSNPs in TRD regions contributed also to cell death and apoptosis {pathways “Cell death signaling via NRAGE, NRIF and NADE” [*P*_(BH)_ = 0.04] and “p75 NTR receptor-mediated signaling” [*P*_(BH)_ = 0.049]}.

### Genetic incompatibilities

**Figure 2:**
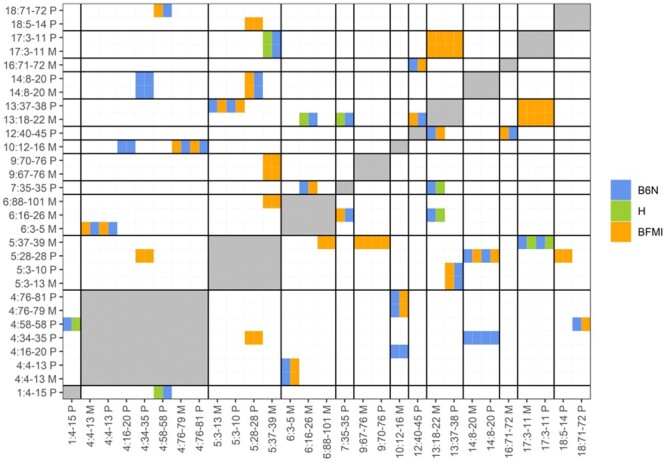
Significant genetic incompatibilities between regions showing TRD. Heat map showing the pairwise genetic incompatibility scan between TRD regions, genome-wide *P*_(BF)_ < 0.05. The allele combination (M1|M2) which is most reduced (in percentages) between the observed and expected allele combinations are shown in the figure with colors denoting the founder allele combination M1 (*x*-axis) and M2 (*y*-axis). Names of regions are composed of chr: start-end allele origin; start and end positions are given in megabase pairs; furthermore, the TRD origin is coded by M for maternal and P for paternal. When two regions were located on the same chromosome the genetic incompatibility test was not performed (gray areas), since the pairwise genetic incompatibility test can only be performed on loci which are not in linkage.

Since functional inaptitude of alleles of interacting pairs of genes could be causal for TRD, we searched for evidence of genetic incompatibilities by a pairwise search between all 62 TRD regions against each other using the Bateson–Dobzhansky–Muller model. This test identifies outliers (significant deviation from Mendelian expectation) between allele pairs by investigating allele frequencies. Severe deviations from Mendelian expectation are interpreted as resulting from negative epistatic interactions between incompatible loci. This search identified genome-wide significant pairwise incompatibilities [*P*_(BF)_ < 0.05] for 29 out of 62 TRD regions (Figure 2), of which 19 were classified as highly significant [*P*_(BF)_ < 0.01]. The high number of TRD regions (46.8%) showing evidence for one or more genetic incompatibilities suggests that genetic incompatibilities are an important contributor to TRD.

Analysis of protein–protein interactions between all 128 protein-coding genes with nsSNPs located in TRD regions showed 331 known interactions between the protein products of these genes. When we ignored genes located on the same chromosome (for which genetic incompatibility tests cannot be performed) we ended up with 273 known protein–protein interactions.

Within the 29 regions that showed genome-wide evidence [*P*_(BF)_ < 0.05] for genetic incompatibilities, 44 out of the 128 (34.4%) protein-coding genes with nsSNPs reside. In total, five known physical protein–protein interactions exist in the STRING database between these 44 genes.

Within these known protein–protein interactions, we found an interaction between alpha-aminoadipic semialdehyde synthase (Aass) located in Mat_R11 (6:16–26 M) with glutathione peroxidase 6 (Pgx6) as well as NME/NM23 Family Member 8 (Nme8) which are both located in Mat_R20 (13:18–22 M). All three of these genes are interesting, given that all three genes are involved in obesity and/or diabetic retinopathy, which are the obvious phenotypic difference between the founders inbred strains BFMI and B6N. The Aass protein is involved in the major mitochondrial lysine degradation pathway (Papes *et al.* 1999; Sacksteder *et al.* 2000) and was found to be downregulated in obese compared with lean cotwins (Heinonen *et al.* 2015)*.* With regard to Pgx6, glutathione peroxidase activity is suppressed in diabetic compared to healthy controls, with a more pronounce suppression in obese compared to nonobese diabetics (Singhai *et al.* 2011). Glutathione peroxidase activity was found associated with diabetic retinopathy (Rodríguez-Carrizalez *et al.* 2014). *Nme8*, encodes an axoneme protein, and mutations in the *Nme8* gene have been implicated to cause primary ciliary dyskinesia (Duriez *et al.* 2007). Furthermore, the genetic region in which the *Nme8* gene is located was identified in human GWAS as a locus which might be involved in childhood obesity in the Hispanic population (Comuzzie *et al.* 2012). *Nme8* is a very interesting gene to come up during this analysis, because of its relation to primary ciliary dyskinesia. The *Bbs7* gene was previously identified as the most likely causal gene for the obesity phenotype of the BFMI mouse (Arends *et al.* 2016). The Bbs7 protein is part of the BBSome complex which is a heterooctameric protein complex that plays a central role in primary cilia homeostasis (Klink *et al.* 2020).

Furthermore, a protein–protein interaction between acyloxyacyl hydrolase (Aoah), located Mat_R20 (13:18-22M) and protein tyrosine phosphatase (PTP) receptor type Z1 (*Ptprz1*) Mat_R11 (6:16–26 M) was found. Aoah is a lipase that plays an important role in the defense against Gram-negative bacterial infection (Lu *et al.* 2008). Aoah^−/−^ mice on a high-carbohydrate diet develop nonalcoholic steatohepatitis and both serum triglyceride and cholesterol were found significantly increased (Ojogun *et al.* 2009). The *Ptprz1* gene is annotated to the insulin receptor recycling pathway, and PTPs are required for the dephosphorylation of the insulin receptor (Fischer *et al.* 1991). Again, both genes identified by our approach are clear candidate genes when considering the major phenotypic differences of the founder strains BFMI and B6N.

The next identified protein–protein interaction between genes with nsSNPs in TRD regions showing genetic incompatibilities was between myosin IE (Myo1e), located Mat_R15 (9:67–76 M) and serine/threonine kinase 32B (Stk32b) Mat_R9 (5:37–39 M). Myo1e is part of the nonmuscle class I myosins which are a subgroup of the unconventional myosin protein family and function as actin-based molecular motor. The *Stk32b* gene is annotated into the “Sweet Taste Signaling” pathway (GeneCards Human Gene Database 2021), and deletion of the gene was associated with Ellis-Van Creveld Syndrome (Temtamy *et al.* 2008) in humans. This interaction does not have a clear link to the phenotypic differences between BFMI and B6N.

Moreover, protein–protein interaction was detected between myosin VC (Myo5c), located Mat_R15 (9:67–76 M) and solute carrier family 2 member 9 (Slc2a9) located in Mat_R9 (5:37–39 M). The Myo5c protein is involved in actin-based membrane trafficking in many physiologically crucial tissues. In humans (and mice), Myo5c is particularly abundant in epithelial and glandular tissues such as pancreas, prostate, mammary, stomach, colon, and lung (Rodriguez and Cheney 2002). *Myo5c* knockout mice show a decrease in total body fat amount and an increased lean body weight [Mouse Genome Database (MGD) 2021; Blake *et al.* 2021]. Its protein interaction partner Glut9 (*Slc2a9*) is part of the SLC2A facilitative glucose transporter family. Members of this family play a role in maintaining glucose homeostasis. Glut9 does not transport glucose, but is classified as a urate transporter. Mutations in the *Slc2a9* gene have been shown to be causal for renal hypouricemia (Matsuo *et al.* 2008; Dinour *et al.* 2010), mice lacking the Glut9 protein show early-onset metabolic syndrome (DeBosch *et al.* 2014).

These known protein–protein interactions between genes in TRD regions with nsSNPs, lead us to hypothesize that disturbed protein–protein interactions resulting from amino acids changes due to nsSNPs within several proteins of a protein complex are likely one of the driving forces causing the TRD observed in the BFMI x B6N AIL.

## Conclusions and discussion

In this study, we examined an AIL population originating from a cross between the obese mouse line BFMI and the standard mouse line B6N in generations 26 to 28 for TRD from parents and grandparents to offspring. The most significant finding of this study was the detection of 62 genomic regions showing TRD in the genotype data from generations 27–28.

We considered three possible explanations for the widespread TRD we observe in our AIL, (1) independent selection at each locus, (2) gametic or meiotic drive, and (3) preferential selection of combinations of alleles at two or more loci.

The first hypothesis that selection happens at each locus independently will often lead to nonproductive crosses and/or massive lethality after birth (Huang *et al.* 2013a). The argument against this first hypothesis is that the litter size in generation 28 does not deviate from the litter size in the parental inbred lines BFMI and B6N (data not shown). When incompatibilities are (embryonically) lethal this would cause a side-effect of significant TRD which should also be detectable as deviation from the HWE in the offspring generation (Paterson *et al.* 2009). Since regions out of HWE were excluded in our study (embryonically), lethal alleles were not investigated in our AIL. This means that the observed TRD cannot be due to lethality, leading us to reject this hypothesis of direct independent selection at each locus as the cause for the TRD observed in our AIL.

The second possible mechanism for the TRD observed in this article are the well-investigated examples in mouse from meiotic drive, such as the *t*-complex (Safronova and Chubykin 2013). In short, meiotic drive can be thought of as a conflict in which a selfish allele is able to use asymmetric meiosis in order to have a greater chance of being transmitted to the gamete. This mechanism in first instance fits our observations well, since pericentromeric regions seem to be involved. Detected TRD regions in our AIL were observed located in close proximity to the telocentric centromere for 10 out of 19 autosomes. This observation is consistent with previous findings in *e.g.*, *Drosophila*, where autosomal meiotic drivers, occur in heterochromatic regions around centromere and telomere (Brand *et al.* 2015). In mice, genetic incompatibilities in and around the centromeric regions between *M.* *musculus musculus* and *M.* *musculus domesticus* have been known for decades (Lenormand *et al.* 1997; Fel-Clair *et al.* 1998) and have been studied extensively in mouse populations near the hybrid zone (Teeter *et al.* 2008; Larson *et al.* 2018). Centromere strength differs between mouse strains and was found to predict the direction of meiotic drive in mice (Chmátal *et al.* 2014). Earlier findings showed no incompatibility between the chromosome 11 centromere region in hybrids between *M.* *musculus musculus* and *M.* *musculus domesticus* (Lanneluc *et al.* 2004). Our study confirms this finding, since we also did not observe TRD at the chromosome 11 centromeric region. Genome-wide DNA sequencing showed that BFMI is a hybrid between *M.* *musculus musculus* and *M.* *musculus domesticus* (data not shown, sequencing data available at SRA). As such, the AIL between BFMI and B6N might have revived incompatibilities stemming from meiotic drive between *musculus* and *domesticus* alleles. However, an argument against meiotic drive causing our TRD is that true meiotic drive would have led to fixation of the favored allele/haplotype within 26–28 generations (Kursel and Malik 2018). Since we do not observe this fixation, the meiotic drive hypothesis is unlikely to underly the widespread TRD observed in our AIL. Additionally, in mammals only female meiosis was found to be asymmetric (Brunet and Verlhac 2011; Kursel and Malik 2018), meaning that our observed paternal TRD is most likely not due to meiotic drive. However, we cannot exclude that this hypothesis might play a role for the maternal TRD regions observed near the centromeric regions. Furthermore, pathway overrepresentation analysis does show overrepresentation of pathways which point to meiotic drive in maternal BFMI TRD regions.

The third hypothesis is that TRD at each locus is not independent but rather caused by selection on preferential combinations of alleles or selection against detrimental allele combinations (Martin-DeLeon *et al.* 2005; Xie *et al.* 2019). nsSNPs in protein-coding genes located in TRD regions were investigated to see if this hypothesis could explain the TRD observed. In total, we found 182 nsSNPs in 128 genes within the 62 identified regions showing TRD. Based on permutation we would have expected to see only 125.9 ± 17.0 nsSNPs. The density of nsSNPs in genes in TRD regions was 44.5% higher than expected by chance. While the changes in amino acid sequence derived from nsSNPs in a single gene might not be sufficient to cause lethality or to reduce fitness, co-occurrence with SNPs in protein–protein interaction partners could cause such adverse effects, *e.g.*, by affecting protein–protein binding leading to signaling problems (Xie *et al.* 2019). Such adverse effects could result in detectable TRD. On evolutionary time scales this is known as protein coevolution, known to leave detectable footprints (Clark *et al.* 2011; Teppa *et al.* 2017). We hypothesize that this is what drives most TRD in our BFMI x B6N AIL. In our AIL, we combine the genome of BFMI (a mix between *M.* *musculus musculus* and *M.* *musculus domesticus*) with B6N, a *M.* *musculus musculus*. Our AIL as such forces together the evolutionary separated genomes of *M.* *musculus musculus* and *M.* *musculus domesticus* which might lead to resurging incompatibilities between proteins coded in different TRD regions.

However, it should be noted that in this article we did not investigate the subspecific origin of the TRD regions and as such we can only hypothesize that protein–protein incompatibilities might be the underlying mechanism for the TRD we observe. Further research on the subspecific origin of the TRD would allow us to disentangle the contribution of each of the three hypotheses on our observed TRD.

Pairwise testing of genetic incompatibilities between the 62 identified TRD regions showed 29 genome-wide highly significant genetic incompatibilities in our AIL. Although our analysis shows that observed allele TRD is likely due to incompatibility between proteins in two or more TRD regions, genetic incompatibilities only account around half of TRD observed. Potentially, some incompatibilities could not be detected since (1) we limited our analyses to pairwise testing TRD regions, (2) incompatibilities might not always lead to detectable TRD, and (3) not all protein–protein interactions are known yet and/or stored in the STRING database. Furthermore, some genomic regions did not contain informative markers, and as such, they did not allow us to test for TRD in these regions. However, we cannot exclude meiotic drive for maternal TRD regions near the centromeres which might act alongside the genetic incompatibility hypothesis.

Pathways overrepresented in maternal BFMI TRD regions strongly point to meiotic drive with pathways such as: “DNA methylation,” “Meiotic/meiosis regulation,” “Chromatin organization,” “Nucleosome assembly,” and “Telomere Maintenance” overrepresented. This is in line with the meiotic drive hypothesis being causal for some of the maternal TRD observed near the centromeres.

Paternal TRD regions showed overrepresentation of “Signaling by Wnt,” “Metabolism of lipids and lipoproteins,” and “Retinol metabolism.” These pathways point to incompatibilities, and genes located in TRD regions, such as acyl-CoA oxidase 2 (*Acox2*), fatty acid binding proteins 4 and 5 (*Fabp4*, *Fabp5*), fatty acid desaturase 2 (*Fads2*), and malic enzyme 1 (*Me1*) which are known to be involved in energy partitioning and metabolic phenotypes in which the BFMI and B6N founders differ. Recent work on the retina of BFMI mice has shown differences in the rhodopsin layer of BFMI *vs* B6N mice, pointing toward an impaired retina function in BFMI mice. Eyes of the BFMI showed definite characteristics of retinal degeneration in terms of a dysfunction of the rhodopsin transport and a reduction in the outer nuclear layer thickness (Brockmann *et al.* 2017). This might explain why genes located in the “Retinol metabolism” pathway come up as significantly overrepresented. Our TRD analysis identifies genes within TRD regions that could be considered as possible candidate genes for retinal degeneration in mice and humans.

When looking into pathways that were overrepresented while analyzing TRD genes with nsSNPs, pathways such as “Cell Cycle,” “Metabolism of lipids and lipoproteins,” “Metabolism,” “Signaling by Rho GTPases” show in both paternal as well as maternal TRD regions. This provides support for the hypothesis that fundamental cell cycle and metabolic processes are affected by TRD and that selection of major phenotypic differences (*e.g.*, body weight and body composition) shaped the allelic composition of the genome of the founder inbred lines by different genetic requirements. These genetic adaptations are necessary for the optimization of the genome to ensure fitness and reproduction during the generation of inbred lines and might be what causes the observed TRD when founder genomes are combined together.

Our study sheds new light on the TRD in a cross between different inbred mouse strains, the distinct functioning of genomes in producing viable offspring, and provides a way to identify candidate genes which could contribute to complex traits different between the founder strains (in our case obesity and/or retinal functionality). The genes in the TRD regions provide new targets for investigating genetic adaptation and modifying determinants of complex traits.

## Data availability

The datasets supporting the conclusions of this article are included within the article and its supplementary files. DNA sequencing data were deposited at the NCBI Sequence Read Archive (SRA) under BioProject ID: PRJNA717237. Supplemental Material included at figshare: https://doi.org/10.25386/genetics.14753628.

## Ethics approval

All experimental treatments of animals were approved by the German Animal Welfare Authorities (approval no. G0016/11).
